# Sex and Gender Influences on Cancer Immunotherapy Response

**DOI:** 10.3390/biomedicines8070232

**Published:** 2020-07-21

**Authors:** Azzurra Irelli, Maria Maddalena Sirufo, Carlo D’Ugo, Lia Ginaldi, Massimo De Martinis

**Affiliations:** 1 Medical Oncology Unit, Department of Oncology, AUSL 04 Teramo, 64100 Teramo, Italy; azzurra.irelli@hotmail.it; 2 Department of Life, Health and Environmental Sciences, University of L’Aquila, 67100 L’Aquila, Italy; maddalena.sirufo@gmail.com (M.M.S.); lia.ginaldi@cc.univaq.it (L.G.); 3 Allergy and Clinical Immunology Unit, Center for the Diagnosis and Treatment of Osteoporosis, AUSL 04 Teramo, 64100 Teramo, Italy; 4 Radiotherapy Unit, Department of Oncology, AUSL 04 Teramo, 64100 Teramo, Italy; carlo.dugo@aslteramo.it

**Keywords:** sex, gender, cancer immunotherapy, immunology, immune checkpoint inhibitors, precision medicine, gender medicine

## Abstract

The global burden of cancer is growing and a wide disparity in the incidence, malignancy and mortality of different types of cancer between each sex has been demonstrated. The sex specificity of cancer appears to be a relevant issue in the management of the disease, and studies investigating the role of sex and gender are becoming extremely urgent. Sex hormones are presumably the leading actors of sex differences in cancer, especially estrogens. They modulate gene expression, alter molecules and generate disparities in effectiveness and side effects of anticancer therapies. Recently immunotherapy aims to improve anticancer treatment strategies reducing off-target effects of chemotherapy and direct cancer cells killing. It is recognized as a fruitful strategy to treat and possible to cure cancer. Immunotherapeutic agents are used to activate or boost the activation of the immune system to fight cancer cells through physiological mechanisms often evaded in the offensive march of the disease. These therapeutic strategies have allowed new successes, but also have serious adverse effects including non-specific inflammation and autoimmunity. Sex and gender issues are of primary importance in this field, due to their recognized role in inflammation, immunity and cancer, and the clarification and understanding of these aspects is a necessary step to increase the responses and to diminish the adverse effects of immunotherapy. This review describes the available knowledge on the role of sex and gender in cancer immunotherapy, and will offer insights to stimulate the attention and practice of clinicians and researchers in a gender perspective of new cancer treatment strategies.

## 1. Introduction

Sex is a biological parameter that influences the development and progression of various diseases, including cancer [1]. Sex and gender are often used interchangeably, but while sex refers to biological characteristics, gender can be defined as roles, behaviors, activities and attributes that a society considers suitable for male versus female from a cultural point of view [2].

In fact, a gender perspective in health should also focus on people’s circumstances in relation to their economic, social, cultural and working conditions. These are significative determinants that may impact on development, diagnosis and response to therapy.

Some authors suggest to use the two words together “sex–gender” in order to grasp the meaning of both the biologic and social context, as sometimes it is difficult to separate gender and sex that are interactive, entangled, multidimensional [3,4,5], but on the other hand the conflation of the two terms can be problematic as in most data sex is reported. Nowadays immunotherapy plays a leading role in the treatment of cancer offering a new perspective in advanced malignancies. Its effectiveness is influenced by numerous factors: the immune response, the intrinsic characteristics of the cancer cell and the environment.

Furthermore, a sex-based dimorphism about its effectiveness is emerging as in other aspects of tumor pathology [6,7]. To optimize cancer treatment strategies, we need to better understand processes that regulate sex and gender immune responses so we can ensure an effective personalized approach.

Prakash et al. evaluated sex representation in phase I, II and III clinical trials, included in ClinicalTrials.gov, with adult subjects enrolled from January 2011 to December 2013 and showed the male prevalence in all studies, including those funded by the NIH [8,9].

However, it must be remembered that it is only since 2014 that the National Institutes of Health (NIH) invited researchers to take sex into account and consider it a biological variable in research. The prejudice that led to the exclusion of females is due to their greater biological variability, mainly justified by fluctuations in sex hormones [9].

In this regard, the role of the European Union (EU) in supporting targeted projects should be underlined [10]. Attention to the aspects of sex in health research has been one of the main initiatives of EU policy; among these the GenderBasic project (2005–2008) [11].

The issue is complex and data are fragmentary and sometimes discordant; in some cases there are only epidemiological observations, while in others the pathogenetic hypotheses are only speculative. In this attempt to give a sex and gender vision of this topic we summarize the knowledge on the sex driven immunological mechanisms involved and then analyzing by immunotherapy type, the facts inferable from the literature, from time to time we will deal with epidemiological data, experimental findings, clinical observations and therapeutic results. Unfortunately, sometimes the result may be just listing conflicting published data and other times just a speculative discussion. Taking stock of the current knowledge, we aim to stimulate further research as the essential step for better development in cancer immunotherapy of personalized and patient-centered care.

## 2. Cancer Susceptibility and Development

### 2.1. Sex–Gender Differences in Cancer

According to the Italian Cancer Registry (AIRTUM), one in two men and one in three women have an average life risk of developing cancer, while one in three men and one in six women have an average probability of dying from cancer [12].The cause should be found in the complex interaction between sex hormones, sex chromosomes, cancer cells, the tumor microenvironment and the immune system [13]. Female cells have shown greater ability to overcome cellular stress through the induction of protective mechanisms, such as autophagy [14], and more antioxidant defenses than male cells [15].

A number of studies have been published that compare patients based on their sex: studies that compare the germline DNA of cancer patients with that of healthy people (controls) to identify single-nucleotide polymorphisms (SNPs) associated with the risk of cancer occurrence; gene expression studies that use microarray or RNA-seq to compare the expression patterns of sex chromosome genes; studies identifying sex-dependent somatic alterations (e.g., copy number gain or loss or somatic mutations) [16,17]. Several gender related risk factors, such as smoking and other habits (alcohol intake, sunlight exposure), environmental exposures (occupational toxins), body weight, dietary patterns and physical activity behaviors have a different impact on men and women in this context. For most types of cancer, males show a higher risk of malignancy during their lifetime than females and have a worse prognosis [18]. Males have an almost double risk of mortality for all malignancies compared to females, particularly for larynx, esophagus, bladder and lung cancers [19]. This higher mortality for the male population reflects not only the differences in the etiology of cancer but also the sexual differences in hormonal regulation and immune system function [20]. Females have stronger innate and adaptive immune responses than males, reducing the risk of cancer mortality. These differences are due to epigenetic and genetic factors, sex hormones and to psychosocial factors [21]. Genes with critical roles in immune response regulation such as those encoding for IL-2 receptor gamma subunit, toll like receptor (TLR)-7, TLR8, CD40L and the forkhead box P3 (FoxP3) are located on the chromosome X. Sex hormones modulate the differentiation, maturation, lifespan and effector functions of innate immune cells including dendritic cells, neutrophils, natural killer cells and macrophages and on B and T lymphocytes. Sex chromosomes and hormones influences self-renewal of systemic determinants of carcinogenesis, stem cell populations and the tumor microenvironments. Women suffer more from autoimmune and inflammatory diseases as a consequence of mounting a more vigorous immune response. Women have a Th1 biased immune system. Sex hormones have been shown to influence the regulation of Th cell network balance in different ways, T helper1 (Th1) and Th2 cytokines (Th1/Th2) have pivotal roles in the homeostasis of Th1 and Th2 cell network functions in the immune response. For immune response homeostasis, the interleukin- (IL-) 6 production pathway is specific to women and the interferon (IFN)*γ* production pathway is specific to men. Common to both but controlled by the respective gender-specific pathways for restoring immune system resting homeostasis is the IL-10 pathway [22]. In mice, adult females produce higher levels of T helper 1-type cytokines such as IFNγ than males, but the Th1–Th2 dichotomy may not always hold true in human males and females. Females in post-puberty adulthood show higher CD4/CD8 ratios and CD4+ T lymphocytes, increased T cell activation and proliferation, lower CD8+, Treg and NK cells. B cells and immunoglobulins are also increased in human females [23].

Except in some cases [24,25] Th1 cells, through their immune functions, can overall be considered, beneficial to induce an efficient antitumor immune response.

As mentioned above, the Th1 phenotype plays a leading role in the development of an efficient antitumor immune responsethrough varying ways, and in particularlybyinducing the stimulation of CTL activity.It is supposed that PDL1 may affect Th1 plasticity. Th1 phenotype preservation could be obtained directly with PD1 blockade, which show significant clinical advantages against cancer. Therefore, an intriguing strategy to fully restore Th1 phenotype could be the adoptive transfer of Th1 cells with an anti-PD1 blocking antibody [26].

Cancer in females must evade more efficient immune surveillance mechanisms and undergo a more intense immune-editing process to become metastatic. This ability of tumors in females to evade immune surveillance makes metastatic tumors less immunogenic and enriched with more efficient immune escape mechanisms and may therefore exhibit resistance to immunotherapy [27,28].

### 2.2. Viruses and Cancer

About 10–15% of human cancers are caused by viral infections and currently available vaccines effectively prevent infection and neoplastic disease.

Vaccines are known to exploit humoral immunity. A difference in vaccination response between the two sexes could be due to the higher levels of CD4 + lymphocytes and to the production of Th1 cytokines, after immunization, in women. It has been observed that higher seroconversion rates in women vaccinated with anti-hepatitis B virus (HBV) may result in a reduced prevalence of development of liver cancer [29].

Human papillomavirus (HPV)-related diseases (including oncological diseases) occur with sex–gender differences. A different inflammatory reaction to HPV is observed in females and males: the estrogen inhibition and the testosterone activation make viral clearance faster in men. HPV affects the genital organs differently: the cervix is most affected in women while in males the genital area is rarely involved. Furthermore, the different behaviors have an effect on epidemiology, making, for example, some groups of men (homosexuals, human immunodeficiency virus (HIV)-positive, smokers, alcoholics) at greater risk of tumors in sites such as the oropharynx and the anus. Socio-economic conditions influence the gender distribution of HPV-related diseases [30]. On the other hand, viruses also represent a therapeutic opportunity. In fact, since 2015 oncolytic viral therapy was approved by the Food and Drug Administration (FDA) that is based on selective infection and the replication of genetically engineered viruses in cancer cells to induce their immune-mediated death.

## 3. Sex, Gender and the Immune System

The immune system differs between males and females, with differences modulated by: genetic mediators such as sex chromosomes (X, Y), hormonal mediators such as estradiol, progesterone and androgens, environmental mediators such as the microbiome [2], social sex behaviors (e.g., smoking and alcohol consumption) [16,17] and age [2].

The random inactivation of an X chromosome in each female cell leads to mosaicism and, in turn, to the advantages associated with genetic heterogeneity. Inactivation balances the expression of X-linked genes but a significant number of genes escape this process. The presence of mutations in tumor suppressor genes on a single allele, retaining two functional copies, could represent a protective mechanism [31].

The X chromosome is linked to immune-related microRNAs whose deregulation has been associated with the pathogenesis of many types of tumors, about 120 microRNAs on the X chromosome, in contrast to the four found on the Y chromosome and with the 40–50 found on average on the autosomes. MicroRNAs act as post-transcriptional regulators of gene expression. Although the role of most of them has not yet been described, microRNAs located on X chromosomes have functions in immunity and cancer. The unique mode of inheritance of the X chromosome is ultimately the cause of the immune disadvantage of males versus females [32].

### 3.1. Sex Hormones and Immunity

Sex hormones modulate the interaction between genes and the immune response: progesterone has extensive anti-inflammatory effects; androgens suppress the activity of immune cells; and estradiol improves cell-mediated and humoral immune responses.

Stronger innate and adaptive immune responses characterize females respect to males. They show a better response to different types of vaccination and are less susceptible to several types of infective agents.

Females produce more vigorous humoral immune reactions than males; estrogens stimulate plasma cells to produce immunoglobulins. Peripheral blood mononuclear cells (PBMCs) from healthy male and female subjects secrete more immunoglobulins when cultured in vitro with 17-beta-estradiol (E2), but their proliferation rate and viability are not affected. E2 can increase the production of human PBMC immunoglobulins mainly by increasing the monocyte release of IL-10, which in turn triggers the B cells secretion of IgG and IgM [33,34].

Estrogen treatment increase the frequency of IL-6 and IL-10-secreting cells in an animal model [35].The treatment of bone marrow-derived dendritic cells (BMDC) with increasing concentrations of dihydrotestosterone (DHT) but not 17-β-estradiol (E2) induce progressive decrease of IL-6 production while levels of IL-10 upon exposure to low hormonal stimulation initially decreases and then increases as DHT and E2 concentrations increase [36].

**Figure 1:**
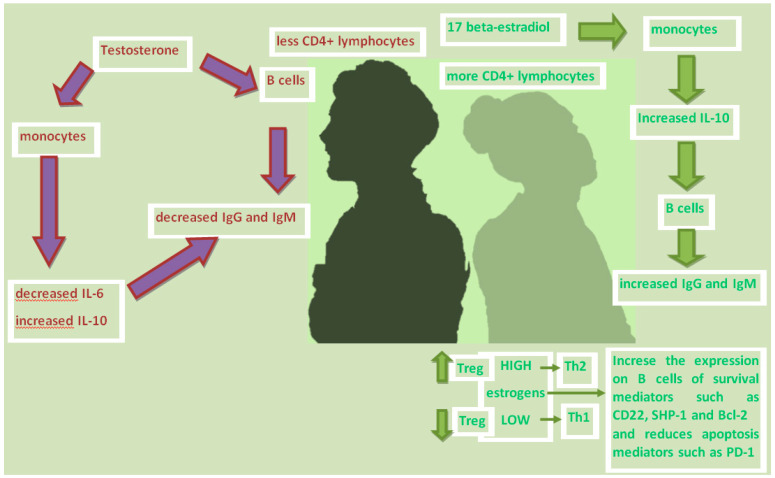
Showing some of the differences between males and females’ immune response. Estrogens increase the production of immunoglobulins mainly by increasing the production by monocytes of IL-10, which in turn triggers the secretion of IgG and IgM by B cells; in contrast, testosterone has been found to reduce immunoglobulin production directly damaging the secretion of IgG and IgM in B lymphocytes, and indirectly by reducing the production of IL-6and increasing IL-10 from monocytes; estrogen increase the expression regulation of B cell survival mediators, such as CD22, SHP-1 and Bcl-2, and change the expression of the PD-1, an apoptosis mediator; T-regulatory cells (T-reg) are sensitive to changes in sex hormone levels during the ovarian cycle: increase with high estrogen levels and decrease with low estrogens and high progesterone; low estrogens means T helper (Th) differentiation towards Th1 while high doses of estrogen towards the Th2 phenotype.

In contrast, testosterone has been found to reduce immunoglobulin production by more than 50%, but acting with a mechanism other than estrogen. Testosterone directly damages the secretion of IgG and IgM in B lymphocytes, and indirectly reduces the monocytes production of IL-6 [37] while increases the anti-inflammatory cytokine IL-10 [38] (Figure 1).

Estrogen has also been shown to directly increase the expression of B cell survival mediators, such as CD22, SHP-1 and Bcl-2, and the alteration of B cell apoptosis mediators, such as PD-1 [39].

Not only B lymphocytes, but also dendritic cells (DC), macrophages, neutrophils and natural killer cells (NK) are sensitive to sex hormones [40].

T-regulatory cells (T-reg) are sensitive to changes in sex hormone levels during the ovarian cycle (Table 1). The frequency and number of T-regs increase during the follicular phase due to the increase in estrogen levels, while they decrease during the luteinic phase in which estrogen is low and progesterone is high. Since T-regs control the expansion of the peripheral T cell pool, and play a central role in maintaining self-tolerance by suppressing self-reactive T cell clones, it can be assumed that the effect of sex hormones on T -reg contributes to the onset of autoimmune diseases in women. Estrogen also selectively controls the expression of some chemokine receptors on T cells. In the case of the CC-chemokine receptors, the CCR5 and CCR1, on CD4 + T cells are stimulated by estrogen. This has consequences on the migratory capacities of reactive T cells not only in the context of infection, but also in autoimmunity. The mechanism by which estrogen affects T cell biology has not yet been fully elucidated. Low estrogen tilts the T helper (Th) response towards differentiation in Th1 enhancing cellular immunity, while high doses of estrogen unbalance the differentiation of Th towards the Th2 phenotype, strengthening humoral immunity.

Estrogen also exerts its repressive effect on the innate immune system. One of the main activating receptors expressed by monocytes is Fcγ RIIIA. Estrogen signaling represses the transcription of the Fcγ RIIIA gene, thereby reducing the ability of monocytes to secrete IL-1β, IL-6 and TNF. Furthermore, the production of these cytokines has been shown to be reduced during the follicular phase of the ovarian cycle (when the estrogen concentration is higher) and increases in the lutein phase (when the estrogen concentration is lower). The phagocytic activity of neutrophils and macrophages is higher in females than males. As described in some studies the number of neutrophils fluctuates during the female menstrual cycle: they decline during follicular phase and increase in luteal phase. They increase significantly in pregnant women when both concentration of estrogens as well as progesterone increases. A dose-dependent effect of estradiol on degranulation was described differently on the release of β-glucuronidase and lysozyme or myeloperoxidase from cytoplasmic neutrophils granules. In addition, estrogens seem to impact the oxygen-dependent intracellular mechanism of killing, but available studies are ambiguous [41,42].

The cytotoxicity of natural killer (NK) cells is also mitigated by estrogens, which down-regulate the expression of NK cells. Estrogens also have a significant impact on the differentiation and activation of dendritic cells (DC) through the production of the IFN regulatory factor (IRF) -4 in myeloid progenitors [43,44].

Estradiol (E2) levels are variable during the menstrual cycle and in the life course, high during pregnancy and low after menopause. Estrogen receptors are expressed in lymphoid tissue, including lymphocytes, macrophages and dendritic cells. E2 improves both mediated and humoral immune responses. Progesterone, another sex hormone, is produced by the corpus luteum during the menstrual cycle and in large quantities by the placenta during pregnancy and has been observed to have broad anti-inflammatory effects. Progesterone receptors are present on many different types of immune cells, including natural killer cells, macrophages, dendritic cells and T cells. Finally, androgens, including dihydrotestosterone and testosterone, which are present in higher concentrations in post-pubertal males than females, generally suppress the activity of immune cells, which means that the immune response is reduced in males [45].

### 3.2. Immunity, Microbiome and Aging

The impact of the microbiome on immunity is recognized. However, the relative contribution of the microbiome is difficult to define since its composition could be influenced by sex in a body-mass dependent way. Sex hormones, in particular androgens, seem fundamental in shaping the composition of the intestinal microbiota [2] that also may change as a consequence of both diet and antibiotic use. Age-related gastric atrophy as well as age-related vaginal changes after menopause affect the respective microbiota. Bacteria can metabolize sex hormones, through the activity of hydroxysteroidal dehydrogenase enzymes that regulate the balance between active and inactive steroids [46].

Aging has a significant impact on estrogen and androgen levels in both boys and girls. While estrogen levels only decrease in menopausal women, there is a gradual decrease in androgens in both sexes starting at the age of about thirty [47,48,49]. Furthermore, the increased risk of malignancy associated with aging is related to the senescence of stromal fibroblasts and the activation of cancer associated fibroblasts (CAF). CAFs are also under the control of the sex hormone, with variable results depending on the tissue [2].

### 3.3. Sex, Genes, Immunity and Cancer

Many genes on the X chromosome regulate immune function, but also the Y chromosome contains regulatory response genes [17].

Immune-related genes on the X chromosome encode proteins involved in the regulation of innate immunity, such as Toll-like receptors (TLR), and in the regulation of adaptive immunity, such as cytokine receptors and transcription factors. These genes encoded on the X chromosome can escape X inactivation, causing higher expression levels in females than in males. Sex hormones are an important determinant of sexual differences in immunity, as they modulate the development and function of multiple populations of immune cells [50]. They exert powerful effects on the regulation of a large number of immune-related genes. Androgen response elements (ARE) and estrogen response elements (ERE) are promoters present on several genes related to innate and adaptive immunity, suggesting that sex hormones can directly regulate the expression of factors influencing immunity [51]. Female cells appear to have more efficient epigenetic machinery than the male counterpart. In particular, the X chromosome contains a high number of non-coding microRNAs (miRs), currently 118, compared to only two miRs located on the Y chromosome and an average of 40–50 on the autosomes. The regulatory power of miRs is well recognized, as 30–50% of all protein-coding genes are targeted by miRs and their role in cell fate has been well demonstrated [52].

Male (XY) and female (XX) cells respond dissimilarly to stimuli probably because of their different ability to cope with cellular stress. This diversity is probably due to the greater ability of XX cells to prevent and repair damage compared to XY cells.

Sexual variations exist in biological DNA repair mechanisms. Although a higher level of DNA damage is found in males, the DNA repair capacity in females is lower [53,54,55]

Furthermore, it has been shown that oxidative stress biomarkers are higher in males than females of the same age [56] and reactive oxygen species (ROS) production is higher in male cells than in female cells. Women seem to be less sensitive to oxidative stress than men [57]. The female immune system appears more efficient in a number of species, including humans [58].

The aromatase enzyme (CYP19A1) converts androgens to estrogens, that by interactions with one of the two receptors, the estrogen receptor α (ERα) or the estrogen receptor β (ERβ) exert both genomic and non-genomic biological effects. The two isoforms of ER are encoded in separate genes but show similar interaction with endogenous hormones. ERs are found both in the nucleus and in the cytoplasm of cancer cells, allowing transcriptional regulation of genes involved in cell survival, proliferation and crosstalk. In particular, E2 appear to play a significant role in the development and malignant progression of several types of tumors, including breast, prostate, endometrium, ovary, colon and lung tumors [59].

### 3.4. Other Factors Influencing the Immune System and Response to Immunotherapy

The immune system is influenced by genetics and sex hormones but also by the interaction between tumor cells, stromal cells and extracellular molecules within TME. The idea that the improvement of tumor immunogenicity and the inhibition of immunosuppressive mediators could suppress the progression of malignant tumors, led to the development of immunotherapies.

Based on its immune characteristics, cancer may be identified as immunosuppressed, immune excluded and immune inflammatory type. These features together with the tumor microenvironment, including mediators and signaling pathways and its acidic and hypoxic conditions, the type and number of intestinal flora, the intratumoral heterogeneity of neoantigens, the tumor mutational burden and several mechanisms of drug resistance in immunotherapy may affect the response to treatment. Cancer cells can recruit immune regulatory cells, release immune inhibitory factors, down regulate tumor antigen expression to escape the recognition and attack of the immune system. They can selectively amplify molecules that evade immunity, including PD-L1, arachidonic acid lipoxygenase and indoleamine-2,3-dioxygenase (IDO) 1 and 2.

Furthermore, as also mentioned above, the gut microbiome is emerging as a modulator of the response to immune checkpoint inhibitors. Currently modest evidence suggests pure physiological sex driven changes in the composition of microbiome, while several data indicate the interaction of gut microbiome dependent metabolites with relevant biological pathways controlled by sex hormones such as Toll-like receptor and flavin monooxygenase signaling [60].

## 4. mTOR as a Link between the Immune System and Sex Hormones

mTOR (mammalian target of rapamycin) is a protein that takes part in the formation of two complexes called mTORC1 and mTORC2. mTORC2 controls cell survival, proliferation and senescence, while mTORC1 regulates cellular metabolism, in particular protein synthesis and the use of glucose [61,62,63,64].

Inhibiting mTOR means increasing immunosurveillance [65,66], as mTOR modulates the relationship between the tumor microenvironment, which includes the immune system cells (macrophages, natural killer cells, neutrophils, helper T lymphocytes, cytotoxic T lymphocytes, regulatory T lymphocytes), and cancer cells [62,67].

The inhibition of mTOR prevents the correct functionality of the natural killer cells [44]. In addition, mTOR is a regulator of the differentiation of cytotoxic T lymphocytes. Blockade of mTOR inhibits the proliferation of regulatory T lymphocytes with minimal effect on helper T lymphocytes and cytotoxic T lymphocytes. In addition, mTOR modulates the role of PD-L1 [67].

Activated mTOR, through mTORC1, induces the activation of S6K1 with consequent phosphorylation of the ribosomal protein S6 (S6rp) with increased translation of mRNA and cell proliferation. The S6K1 can also be activated by the Ras/MEK/MAPK cascade. S6K1 can activate ER by phosphorylation, leading to ligand-independent activation. Furthermore, phosphorylated ER can in turn activate S6K1 [68,69,70].

17*β*-estradiol (E2) promotes downstream activation of PI3K-mTOR signaling and hepatocyte proliferation through the G-protein–coupled estrogen receptor 1 (GPER1), thus GPER1 promote the E2 mediated initiation and progression of liver carcinogenesis. A sexually dimorphic mTOR activity in the liver was reported in previous researches [71,72] and Chaturantabut et al. postulated a sex dimorphic fashion through GPER1 mediated E2 activation of PI3K-mTOR in liver re generation [73] The androgen receptor (AR) pathway cross-talk with the PI3K/Akt/mTOR and with other receptors such as estrogen receptor and human epidermal growth factor receptor-2 [74]. Chen et al. showed that the suppression of androgen and PI3K/Akt signaling, results in prostate cancer (PCa) cell proliferation [75]. PCa growth and survival is supported by cellular metabolism reprogramming through AR signaling. In fact, androgen-induced aerobic glycolysis and mitochondrial respiration need activation of mTOR-dependent metabolic gene networks after an AR dependent reprogramming of mTOR-chromatin associations [76].

## 5. Sex and Effectiveness of Cancer Immunotherapy

Immunotherapy is an innovative approach to cancer with the aim of modulating the immune system to attack, in multiple directions and targets, cancer cells. It is now acknowledged its incomparable advantage to prolong progression free survival and overall survival. Tumor immunotherapy can be performed in different ways: cellular immunotherapies (adoptive cellular immunotherapy (ACI), NK cell therapy, chimeric antigen receptor T-cell (CAR-T) immunotherapy, immune checkpoints inhibitors (PD-1 ICIs, PD-L1 ICIs, CTLA-4 ICIs), oncolytic viruses, vaccines (tumor cell vaccines, genetic vaccines, dendritic cell vaccines, protein/peptide-based vaccines, in situ vaccines, neoantigen vaccines) and others (targeting myeloid derived suppressor cells, cytokine gene therapy, targeting tumor microenvironment, oncolytic peptides). All these therapeutic strategies open considerable prospects in the fight against cancer, some still take the first steps while others have already demonstrated their efficacy and safety and are now included in the current therapeutic protocols. It is a field in constant renewal and in which the gender perspective is still too often overlooked. Moreover, males and females metabolize certain drugs such as rituximab, at different rates and this could lead to different response rates [77].

### 5.1. Immune Checkpoint Inhibitors

Immune checkpoint inhibitors (ICI) represent a milestone of immunotherapy, they have a promising future in cancer treatment and a variety of ICIs have been approved for the treatment of several types of malignancies. However, only some subjects benefit from the use of these molecules in monotherapy. Therefore, it is necessary to better understand differences in efficacy and adopt integrated therapeutic strategies to increase responses and diminish side effects [78].

Immunotherapy with immune checkpoint inhibitors (ICIs) that simply enhances immune response may result less effective in women than in males, where it is already high. Furthermore, the increased antigenicity in male cancers explains why immunotherapy with ICIs alone is more effective in males.

On the other hand we should consider that tumors with a high tumor mutational burden (TMB) are highly immunogenic, and such tumors exhibit stronger immunosuppressive signals in women than in men so that in similar cases, as ICIs block immunosuppressant signals between cancer and immune cells, they could have a better therapeutic effect in the former [20].

Therefore, different strategies for the treatment of cancer should be proposed in the two sexes: we should improve the immune environment in male patients and the antigenicity of the tumor in females. Overall, ICIs are more effective in male patients than in female patients, while ICIs in combination with chemotherapy are more effective in women than in men [20] (Table 2).

In the meta-analysis performed by Botticelli et al. was reported a trend of greater, not significant, benefit of treatment with ICI in male patients [79]. These results were confirmed by Pinto et al. in patients with non-small cell lung cancer: anti-PD1 inhibitors significantly improve progression-free survival (PFS) in male patients compared to chemotherapy (hazard ratio (HR) = 0.76; 95% confidence interval (CI) 0.68–0.86) while females showed no benefit (HR = 1.03; 95% CI 0.89–1.20) [80]. Conforti et coll. also reported a greater overall survival benefit in males with ICI treatment (ipilimumab, tremelimumab, nivolumab or pembrolizumab). Aggregate overall survival was 0.72 (95% CI, 0.65–0.79) in males and 0.86 (95% CI, 0.79–0.93) in females treated with ICI compared to control. The difference in efficacy between the two sexes was significant (*p* = 0.0019).

**Table 2:** Clinical outcome with immunotherapy according to the sex of patients (overall survival (OS), hazard ratio (HR), progression-free survival (PFS)).

Meta-Analysis	Sex	OS-HR (anti-CTLA4)		OS-HR (anti-PD1)		PFS-HR (anti-PD1)		OS-HR (anti-CTLA4 + anti-PD1)	
			*p*		*p*		*p*		*p*
Botticelli et al. [70]	*M*	0.65	0.078	0.72	0.285	0.66	0.158		
	*F*	0.79		0.81		0.85			
Pinto et al. [79]	*M*					0.76	0.69		
	*F*					1.03			
Conforti et al. [28]	*M*							0.72	0.0019
	*F*							0.86	
Wallis et al. [81]	*M*							0.75	0.60
	*F*							0.77	

ICIs can improve overall survival in patients with advanced cancers, such as melanoma and non-small cell lung cancer, but the extent of the benefit depends on sex [28,29].

On the other hand, a mouse study reported a different trend showing that PD-L1 blockade is more effective in treating melanoma in females than in males. This was partially due to the increased ability of anti-PD-L1 antibodies to reduce Treg function in female mice [59]. Preclinical animal studies also suggest that sex hormones regulate the expression and function of PD-1, and that hormonal effects mediated by PD-1 modulate autoimmunity. The expression of the PD-1 ligand, PD-L1, was then shown to be modulated in an estrogen-dependent and sex-dependent manner [28].

PD-L1 is an immunomodulator expressed by 20–50% of tumors, as well as on stromal cells of the microenvironment. The PD-L1/PD-1 interaction (present on cytotoxic T lymphocyte) is inhibitory: the cytotoxic T lymphocyte does not activate. The cytotoxic T lymphocyte is capable of producing INF-gamma, which acts on a receptor present on the tumor cell by increasing the expression of PD-L1 and PD-1. Hence the cancer cell itself induces immunosuppression.Wallis et al. [81] showed no significant sex differences in the clinical benefits of ICI. An overall survival benefit of immunotherapy was found for both males (HR = 0.75; 95% CI, 0.69–0.81) and females (HR = 0.77; 95% CI, 0.67–0.88) and the difference between the two sexes in response to ICI was not statistically significant. However, this research contained four studies that tested the combination of ICI plus chemotherapy. This may explain why they failed to identify unlike Conforti, a statistically significant difference between the two sexes in terms of ICI effectiveness [28].

A new meta-analysis by Conforti and colleagues showed how females achieve greater clinical benefits from anti-PD1/anti-PD-L1 plus chemotherapy compared to control treatment versus males: females combined a overall survival risk ratio (OS-HR) = 0.44 (95% CI, 0.25–0.76), while males grouped OS-HR = 0.76 (95% CI, 0.64–0.91). The previous observation that males derive greater clinical benefits from ICI alone compared to control treatment in respect to females was also validated: females grouped OS-HR = 0.97 (95% CI, 0.79–1.19), while males grouped OS-HR = 0.78 (95% CI, 0.60–1.00) [51].

The response heterogeneity must also be sought in the ability of chemotherapy to increase the mutational load of female tumors. A different efficacy of chemotherapy could be hypothesized in modulating the anti-tumor immune responses between the two sexes, given the differences related to sex in the quantity and composition of the reported intratumoral immune infiltrates.

The reason why the combination strategies of chemotherapy and ICI show a greater benefit in females may be that chemotherapy can increase the mutational load of tumors and therefore the antigenicity of cancer cells, and females are able to eliminate these highly antigenic tumors more efficiently than males [28]. Recently, the anti-estrogen fulvestrant has been identified as the ideal candidate to combine with anti-PD-1/PD-L1 agents, thanks to its non-overlapping toxicity profile [54].

In the study by Hassler et al., both males and females with metastatic renal cell carcinoma had an OS and PFS advantage with immunotherapy, but no statistically significant difference between the sexes was observed [82]. These conflicting results may be due to the sample size or an intrinsic difference in the etiology of cancer [28].

There was substantial heterogeneity between studies on the extent of effectiveness of ICI in male patients, but not in females. This heterogeneity could be explained by the large number of tumor histotypes, treatment lines and types of treatment analyzed. Other factors, specific to each tumor histotype, type and treatment setting, are likely to contribute, together with the sex of the patients, to the extent of the effectiveness of the ICI. In a recent study on melanoma patients receiving anti-PD-1 therapy, significant differences were found in the composition of the gut microbiome of responders compared to non-responders. Patients with the most diverse microbiome were more likely to respond to immunotherapy, while antibiotic therapy induced resistance to the anti-PD-1 block [48].

In conclusion, ICIs can improve overall survival in patients of both sexes in some advanced cancers, such as melanoma and non-small cell lung cancer. The benefit is largely dependent on sex, with increased efficacy in in males in all types of cancer, regardless of lines of therapy [28]. Furthermore, the molecular evaluation of the specific tumor should be considered. EGFR-mutated non-small cell lung cancers are significantly less sensitive to ICI than wild-type EGFR-type non-small cell lung cancers and are more common in female than in male patients. The BRAF mutation, which is the most relevant genetic alteration in the pathogenesis of melanoma, has never been reported as a predictor of the effectiveness of ICI and is not distributed differently between the two sexes. ICIs can significantly improve overall survival in patients of both sexes, the extent of this benefit is largely dependent on sex, but not only [83,84].

It is unknown whether the better efficacy of immune checkpoint inhibitors in males with non-small cell lung cancer than in females is linked to their smoking history or gender. In addition, oncogenic mutations, such as EGFR or ALK mutations, are known to be more common in women than men and are also known to predict a reduced benefit from immune checkpoint inhibitors. Conforti and colleagues point out that most of the women included in their analyzes had non-mutated EGFR and unresolved ALK tumors [28,50].

The tumor mutational burden (TMB) shows an improvement in performance compared to PD-L1 in predicting response to ICI. In non-small cell lung cancer, the performance of TMB, but not the PD-L1 expression in predicting the ICI response, is significantly better for female than for male patients.

The predictive power of PD-L1 expression on ICI response is not affected by patient’s gender. Probably because PD-L1 expression is directly involved in the function of ICI and, consequently, the predictive power of PD-L1 expression is not influenced by sex differences [84].

The main trend is that different risk factors may have a different distribution between the sexes. The expression of the PD-L1 protein is the most used marker to predict the response to checkpoint inhibitors, despite the heterogeneity of the results observed with the various immunohistochemical methods. A different expression PD-L1 between the two sexes has not been recorded.

The association between obesity and ICI efficacy has been driven mainly by males, and high muscle mass may be one of the reasons responsible for the difference in survival based on sex [85,86].

Females with non-small cell lung cancer appear to be less likely to be smokers than men, and it is also well acknowledged that smokers with non-small cell lung cancer are more likely to benefit from immune checkpoint inhibitors. This could be due to the fact that smokers are less likely to carry oncogenic driver mutations, and that oncogene-addicted cancer is less responsive to immunotherapy [87,88,89,90]. Some of the patients with driver mutations may derive less benefit from immune checkpoint inhibitor approaches either alone or combined with chemotherapy.

To improve patient outcomes in advanced NSCLC it becomes essential the identification of the molecular subset, in fact the presence of actionable driver mutations may significantly impact the choiceofthe better immunotherapeutic treatment [91].

The KEYNOTE-045 study reported a greater benefit of pembrolizumab in current smokers than non-smokers and, similar to the previously described considerations for non-small cell lung cancer, men are more likely to be current smokers than women [89].

The findings also open opportunities to identify new therapeutic approaches for other cancers such as breast cancer, in which the first effective immunotherapeutic approach including a checkpoint inhibitor (atezolizumab) combined with chemotherapy for women with advanced triple negative breast cancer has been described in the IMpassion 130 study [90].

Wang et al. [7] studied the sex-based variances of several genomic immune related factors that could affect the immunotherapy response. They showed, for a pan cancer analysis, that there is no significant sex related difference in TMB while neoantigen burden, tumor purity, cytolitic activity and CD8+ T cell are significantly higher in male subjects than in females. Only CD274 (PD-L1) expression among immune checkpoint related genes is significantly higher in males. So far, we have described several contradictory reports on the results of ICIs in advanced cancer respect to being male or female, and it seems clear that further evidences necessitate to clarify the controversy on sex membership discrepancy. There is heterogeneity among different types of cancer in sex-based differences that influences ICI benefit and sex disparities of their efficacy may be the result of an integrated function of multiple immune related factors. Immunotherapy efficacy may vary between females and males, on the basis of sex biased immunogenomic differences [7].

### 5.2. Vaccines and Other Therapies

Other than ICIs, there are several types of immunotherapy all aiming to hit and kill cancer cells: cancer vaccines, monoclonal antibodies, adaptive cell therapy, chimeric antigen receptor T cell therapy [92]. The advantage of active immunotherapy, which is a relatively new and promising strategy, could be in urging the immune system (IS) to reactivate and support an effective anti-tumor surveillance. The foundation of vaccination consists in the ability of the IS to recognize overexpressed and/or abnormal antigens on the surface of tumor cells. Personalized cancer vaccines include a wide array of approaches including whole tumor cells, protein, peptides, viral vectors, dendritic and T cells, DNA, RNA, that would induce the IS to lead the war against the specific traits of an individual tumor [93]. The different components of the tumor microenvironment, such as natural killer cells, macrophages, dendritic cells and adaptive immune cells play a leading role in the early identification, eradication and progression of cancer. The most relevant component of antitumor immunity is represented by activation of CD8+ cytotoxic T cells that secrete several cytokines such as interferon gamma and tumor necrosis factor alpha to exert their action. Additionally, CD4+ T cells showed the ability to specifically recognize tumor associated neo-antigens and are able to facilitate cancer clearance and reshape the tumor microenvironment [94]. The number of these families of lymphocytes and their ability to recognize tumor associated antigens influence inhibition of growth and development of malignancies. Cancer cells change their surface molecules, reduce their expression, down-regulate MHC class I proteins, create TCR signaling defects and down-regulate co-stimulatory molecule expression. Further modalities to avoid immune detection are developed through Tregs immunosuppressive tumor microenvironment and stopping activation of regulatory pathways while increasing the production of interleukin-10 and tumor growth factor beta. Sex specific pathways of cytokines control the homeostasis of IS and therefore this also supports the hypothesis of the need for a sex–gender perspective in assessing the right strategy in cancer immunotherapy [95].

It is acknowledged that sex drives most of these mechanisms and actors [23,96,97,98,99,100,101,102,103,104]. However, it is not easy to draw conclusions on how these influences manifest themselves in different types of neoplasms, in individual people and how they interfere with different therapeutic strategies. If it is true that significant differences in the immune response of men and women have been documented, it should also be borne in mind that in patients undergoing immunotherapy, the immune system can be compromised by both advanced disease and previous treatments. Many immunotherapeutic approaches are still in an embryonic phase, there are still few clinical trials, few studies in the literature and few evidence or insights regarding the role of sex and gender in relation to their effectiveness. Data on the impact of sex and even more of gender on immunotherapies other than ICIs are very few, firstly, because there is still no structured approach in this perspective, and because in isolated studies no significant differences emerged between men and women. Older forms of immunotherapy exist such as the bacillus Calmette–Guerin (BCG) vaccine and interleukin-2 that elicit a general inflammatory response. Several types of cancer were successfully treated with BCG immunotherapy [104], the live attenuated vaccine developed against tuberculosis that induces trained immunity. These no specific results of BCG vaccination are sex specific [105].

BCG is also successfully used as an adjuvant in different vaccine approaches to cancer, as described by Pampenaet et al. in cutaneous melanoma patients [106].

The treatment of urothelial carcinoma of the bladder with BCG seem to have a reduced effectiveness in women that compared to men show an increased risk of recurrence [107]. The monoclonal anti-IgE antibody omalizumab is an established treatment in several allergic diseases [108,109,110,111,112,113,114] that appear to be a promising treatment in systemic mastocytosis [115] and show sex differences in its efficacy, tolerability and safety [116,117]. Iwai K et al. studied the autologous activated T lymphocytes therapy in advanced lung cancer and showed a significant additive effect on females with adenocarcinoma of immunotherapy on chemotherapy [118]. Kim SW et al. reported that for patients with relapsed or high-risk diffuse large B cell lymphoma (DLBCL) treated with high dose therapy and autologous stem cell transplantation being a male is an independent adverse factor for survival [119].

Male patients with DLBCL or follicular lymphoma treated with the monoclonal antibody rituximab that targets CD20 on B lympocytes surface have a worse progression free survival than females. Better treatment outcomes are more remarkable in females, the clearance of rituximab is higher and the elimination half-life is longer in males with DLBCL [120]. Similarly, bevacizumab—the monoclonal antibody that blocks vascular endothelial growth factor and inhibits angiogenesis—has a clearance that is higher in males with solid tumors respect to females [120]. Graft versus host disease (GVHD) represents the main obstacle to successful hematopoietic stem cell transplantation. Analysis of data from the European Society for Blood and Marrow Transplantation show that there are more male donors than female and there is a significantly higher proportion of no GVHD with male donors than with female and lower in male recipients. Female recipients are more likely to develop GVHD with female donors and in contrast they are more likely to be protected from GVHD than male recipients [121]. Nakasone et al. suggest that hematopoietic cell transplantation (HCT) and bone marrow (BM) transplantation may result in better outcomes than peripheral blood stem cells (PBSCs) when we use female-related donors for male patients [122]. The variables usually considered in the donor selection for hematopoietic cell transplantation include donor/recipient gender [123]. Furthermore, Takekyo et al. reported that men who have undergone hematopoietic stem cell transplantation have a better physical function and quality of life (QoL) than women [124]. On the contrary, Bhatt et al. reveal that after children’shematopoietic cell transplantation, female sex is associated with poorer health related QoL [125]. Volk et al. reported in an animal model a sex-different T cell reconstitution after cord blood stem cell transplantation: peripheral expansion/activation of memory T cells dominate in males while in females is supported by a higher thymic naive T cell output [126]. Anti-cancer vaccines target antigens whose expression is associated with sex [34,127]. Renal clear cell carcinoma, head and neck squamous carcinoma, lung, liver bladder and colorectal cancers show molecule differently expressed in females and males. Up or down regulation of frequency of gene methylation, activity of enzymes and regulation of proteins and mRNAs are included in these sex specific differences, however, the underlying mechanismsare still unclear. These discrepancies suggest specific sex biased treatments against different types of cancer [128]. Sex discrepant responses in immunotherapy may be due to both sex specific differences in immune function and immune related conditions. These relations and mechanisms are complicated, but their understanding is of clinical relevance because it can indicate when immunotherapy will be effective and when to use it. Following a cancer diagnosis many factors are associated with sex differences in prognosis and we must consider more hypothesis outside of a true biological effect of sex. Several malignancies offer a broad range of examples: from the advanced melanoma showing different degrees of lymphocytic infiltrate to the lung cancer with its somatic mutational burden and prevalence of smoking habit. Immunotherapy may stimulate immune system to recognize neo-antigens that originate from the mutational burden of somatic alterations, higher in the inactivated X chromosome of cancer genomes [129].

## 6. The Role of Gender in Cancer Immunotherapy

Discussing the role of gender in opposite to sex in cancer immunotherapy is complex. It is an interesting and multifaced topic with several confounding and difficult to evaluate factors. The lack of validated tools to assess gender is an additional difficulty that also makes studies hardly to compare. Stress-related disorders could have long-term consequences on health outcomes and represent another important effect of sex and gender. Exposure to persistent stress is associated with increased vulnerability also in cancer, of which may affect the outcome. If we hypothesize a role of the behavioral immune system [130] in the effectiveness of immunotherapy, being this influenced by affective and emotional elements and possessing a more proactive function than the biological system, we expect significant gender differences, which should be examined.

In the literature, this topic is virtually absent and continues to remain an under researched area. As gender is highly context specific and socio-culturally constructed, it is difficult to be observed and measured [131]. General considerations can be made that stimulate specific useful insights for further investigations. It is well established that alcohol consumption, poor diet, limited exercise, and tobacco smoking are related to the increased risk of many cancers, as well as poorer outcomes after diagnosis and that geographic disparities in cancer and cancer outcomes exist. Poorer quality of life and psychosocial wellbeing is also evident for cancer survivors. For example, a well-documented health divide exists between major cities and regional and remote areas in Australia [132]. Populations with larger proportions of older people, social and economic disadvantage, poorer access to health care outside major cities, differences in health behaviors across varying environmental contexts and the participation in cancer screening programs, could explain these geographic disparities and gender may affect all these factors. Gender impacts with all aspects of our health, andis intertwined with our real lives. Social dynamics, health behaviors, cultural and educational aspects, work and work environment are only some variables of this complex matter, all interrelated. Social behavioral models have shown considerable power in explaining mortality and morbidity differences on the basis of gender [133,134,135]. Cancer patients are a specific subgroup of people that encounter severe emotional, existential and physical problems. They need special care in relation to their quality of life, personal aspirations, needs, values and relations. Gender-based differences exist in regard to thinking, memory, solving problems and sensitivity to danger or threat. Significant gender-related differences are reported concerning health care between women and men in relation to confiding in crisis, communication styles, coping with illness-related distress, involvement in medical decision and the request for psychosocial support [136]. Women tend to seek health information and care more than men, and men are less likely to report adverse reactions to therapies [131]. The onset and the course of chronic diseases, included cancer are influenced by lifestyle habits, in turn influenced by gender. Among men and women, different patterns of unhealthy and healthy lifestyles depend on gender behaviors and attitudes. Gender dimorphism is reported in health-related behaviors: eating and dietary intake, physical activities, habits such as smoking and alcohol, personal care and attention to the state of health, occupational state and related conditions, respect for therapeutic prescriptions and use of sun protection. In turn, these attitudes can change in the course of life with age, or following specific personal experiences. Each of these aspects can have further repercussions, for example dietary habits can modify the microbiota [137] and cause obesity and we know how they both affect an individual’s immune status and how they can have consequences on a possible neoplastic pathology, its care and outcome [138]. Strong positive co-associations with increased tumor mutational burden and immune checkpoint inhibitor efficacy was observed as the consequence of exposure to several mutagenic causative factors—such as ultraviolet light for melanoma and tobacco smoke for non-small-cell lung cancer. Subjective feelings such as self-concept, happiness, optimism, the use of coping strategies, family functioning and social support are correlated in cancer patients with better psychosocial quality of life [139] and may differ across gender [140]. Although women suffer from stress-related psychiatric disorders more frequently, paradoxically they seem more capable of coping with a disease such as cancer and favorably influencing its outcome.

Two studies showed a strong association between female gender and psychological distress linked to kidney cancer [141,142,143]. A link between psychological factors, stress and cancer is recognized, and recently Inderberg and Wälchli highlighted the influences on molecular mechanisms taking place during T lymphocytes priming and cancer vaccination of ourpsychological status [144]. This indirectly suggests a link between gender and response to cancer vaccination and in a broader vision to immunotherapy. McFarland reported a sex dependent association of depression in lung cancer patients treated with targeted therapy and immunotherapy in respect to chemotherapy [145].

## 7. Patients’ Sex and Adverse Events of Cancer Immunotherapy

Sex dependent adverse drug reaction in cancer therapy are known [146]; furthermore, it has been observed that females report multiple adverse events with immunotherapy (immune-related adverse events: irAE), in particular endocrinopathies, arthritis and pneumonia, hence a higher rate of treatment discontinuation [28,29].

Higher rates of irAEwere recorded in premenopausal females than in postmenopausal females and males. No difference in irAE grade 3 was recorded between the two sexes, but females were more likely to receive oral or intravenous steroids than males, suggesting that females may be treated differently for immunotherapy complications. One possible explanation for the discrepancy in the treatment of irAE includes the differences in the type of irAE experienced for each sex. Pneumonia was more frequent in females; which is treated with oral or intravenous steroids. On the other hand, males had higher rates of dermatological toxicity, which are usually treated with topical steroids.

Factors such as race/ethnicity, BMI and genetic predisposition to autoimmune disorders, could also be risk factors for the development of irAE.

The use of steroids may decrease the effectiveness of the ICI, as a result of possible suppression of IL-2 and the increase in immunosuppressive regulatory T cells, but data from other studies have suggested that the treatment of irAEs with steroids does not affect the result.Grade 2 or greater irAEs have been associated with better PFS and OS. However, a strong relationship between irAE and efficacy results has not been reported in large prospective studies [147].

Most clinical studies of immune checkpoint inhibitors have excluded patients with known or suspected active autoimmune diseases (with the exception of vitiligo), particularly when immunosuppressant drugs and patients with clinical manifestations that were deemed relevant were recently taken clinic. The association between autoimmunity and the efficacy and safety of immunotherapies remains unclear. Because these drugs can induce a range of serious autoimmune adverse events, pharmaceutical companies have decided to exclude patients at increased risk of adverse events from the studies. Some data from retrospective studies suggest that patients with underlying autoimmune diseases can be treated safely and effectively with immune checkpoint inhibitors. On the contrary, the adverse events induced by the immune system could have a positive prognostic value, as demonstrated by the association between the development of vitiligo and the best responses to immunotherapy in metastatic melanoma. Several ongoing studies are investigating the factors associated with the risk of immune-related adverse events (e.g., germinal genetic background and intestinal microbiota) and their role as predictive markers. The prevalence of autoimmune disorders is highly distorted towards women. It is estimated that 6% of the general population is affected by autoimmune disorders, 80% of which are women. In addition, the onset, severity and outcome of many autoimmune diseases are associated with sex [148].

Contributing factors in explaining differences in adverse reaction between the two sexes are often recognized in gender related factors such as cultural, psychosocial or behavioral differences. Health seeking behaviors, social roles and even gender bias in drug prescribing can lead to differences in perception of adverse reactions. Self-image (or body) for example, may differently influence the perceiving of some adverse reactions in the two sexes [131].

## 8. Conclusions

Sex is defined by sex chromosomes and sex hormone levels and is a variable that influences both innate and adaptive immune responses; however, less than 10% of immunology-related publications analyze their data considering the gender of the patients.

In immunotherapy clinical trials, women are still underrepresented compared to men. This is probably due to the fact that men are often used to represent the human species for historical reasons, and there is a fear that cyclic hormonal changes in a woman’s body may influence the results of clinical trials. However, it would be wrong to assume that the results obtained by male patients apply to female patients and vice versa; therefore, clinical trials on cancer immunotherapy should be focused on detecting sexual differences [16].

Future research should ensure greater inclusion of women in studies and focus on improving the efficacy of immunotherapies in women, perhaps by exploring different immunotherapeutic approaches in both sexes [17].

Prospective studies are needed to improve our understanding of these observations and to determine if there is an association between irAE and response to immunotherapy.

All cancer related issues need more gender attention, in the broadest sense of the term, with the inclusion of lesbian, gay, bisexual, transgender and intersex people, because there is aneed for the achievement of a truly inclusive model of precision medicine, even if including these minorities will not be easy due to their small numbers, as reported by Clarke et al. [149].

Other than sex, the gender-identity metric should become essential for the better understanding and description of cancer and for treatment considerations [150].

## Figures and Tables

**Table 1:** Action of estrogens on several immune parameters depending on their concentrations.

ESTROGENS	
**HIGH**	**LOW**
Increased Treg	Decreased Treg
Th2 differentiation	Th1 differentiation
Reduced monocytes secretion of IL-1β, IL-6 and TNF	Increased monocytes secretion of IL-1β, IL-6 and TNF
Increased chemotactic capacity of neutrophils	Reduced chemotactic capacity of neutrophils
Mitigated cytotoxicity of natural killer (NK) cells	Stimulated cytotoxicity of natural killer (NK) cells
Increased differentiation and activation of dendritic cells	Reduced action on dendritic cells
